# Off-World Mental Health: Considerations for the Design of Well-being–Supportive Technologies for Deep Space Exploration

**DOI:** 10.2196/37784

**Published:** 2023-02-14

**Authors:** Nathan Smith, Dorian Peters, Caroline Jay, Gro M Sandal, Emma C Barrett, Robert Wuebker

**Affiliations:** 1 Protective Security and Resilience Centre Coventry University Coventry United Kingdom; 2 Centre for the Future of Intelligence Cambridge University Cambridge United Kingdom; 3 School of Engineering University of Manchester Manchester United Kingdom; 4 Department of Psychosocial Sciences University of Bergen Bergen Norway; 5 Department of Criminology University of Manchester Manchester United Kingdom; 6 David Eccles School of Business University of Utah Utah, UT United States

**Keywords:** long duration space exploration, astronaut mental health, countermeasures, digital design, human factors, technology

## Abstract

During future long-duration space exploration missions, humans will be exposed to combinations of extreme physical, psychological, and interpersonal demands. These demands create risks for the safety, performance, health, and well-being of both individuals and crew. The communication latency in deep space means that explorers will increasingly have to operate independently and take responsibility for their own self-care and self-management. At present, several research programs are focused on developing and testing digital technologies and countermeasures that support the effective functioning of deep space crews. Although promising, these initiatives have been stimulated mostly by technological opportunity rather than cogent theory. In this perspective, we argue that digital technologies developed for spaceflight should be informed by well-being–supportive design principles and be cognizant of broader conversations around the development and use of digital health applications, especially pertaining to issues of autonomy, privacy, and trust. These issues are important for designing potentially mission-critical health technologies and may be determining factors in the safe and successful completion of future off-world endeavors.

## Introduction

Human spaceflight is characterized by extreme physical and psychological demands, which pose significant risks to survival, performance, and health. Explorations that range farther from the Earth will amplify those demands and associated risks [1,2]. Specific risk areas relevant to future long-duration space exploration (LDSE) missions, such as a crewed mission to Mars, are detailed in NASA’s Human Research Roadmap. NASA and other international agencies agree that a significant risk of LDSE missions is prolonged isolation and confinement. Living and working in isolated, confined, and extreme (ICE) environments can lead to declines in individual mental health and well-being (commonly referred to in space research as behavioral health) and interpersonal relations [3-5]. This disturbed psychosocial function in combination with other environmental risks, such as those associated with radiation and microgravity, can adversely impact individual and team performance and, in the worst cases, endanger the lives of the crew [6,7].

The increased communication latency in deep space means that crew members participating in future LDSE missions will have to operate with increasingly greater independence [8-10], suggesting that the countermeasures used for managing issues related to behavioral health on low Earth orbit (LEO) platforms, like the International Space Station (ISS)—which often rely on real-time communications—may be less useful [11]. Alternative methods to support astronaut health and well-being, particularly methods that do not rely on immediate external input or real-time communication, are likely to play an important role in future LDSE missions [12,13].

New systems to monitor and support astronauts' health and well-being and improve the function of both individuals and crew [14] are often augmented with input from the computer sciences, for example, by leveraging the contributions of machine learning algorithms and agent-based models [15,16]. In this paper, we discuss this emerging area of work and highlight important considerations for the development of digital systems for managing behavioral and mental health in space. We argue that the development of these systems must (1) use psychological theory in design and (2) fully consider ethical issues connected to trust, privacy, and human autonomy. This conversation is already happening in the broader domain of digital medicine [17] and provides important lessons for the development of potentially mission-critical health technologies and hence for the success of future off-world endeavors.

## System Types

### Overview

Broadly, we have observed that digital systems for behavioral and mental health management in space and space-analog settings (eg, ICE environments) comprise 3 categories: (1) systems for individual and team monitoring; (2) digital countermeasures; and (3) integrated applications that combine monitoring and countermeasure functions (see Table 1 below for examples). We will discuss research in these areas in the following sections.

**Table 1:** Digital monitoring systems and countermeasures.

System type and example			Description
**Monitoring**			
	Digital monitoring logs	Digitized version of psychometric standard measures used to assess subjectively rated behavior, health, and performance	
	Cognition tests	Test batteries used to determine cognitive function across various dimensions	
	Optical assessment	Automated assessment of facial features as a way of understanding stress and fatigue	
	Speech and language	Method for analyzing the content of language and the dynamics in speech to understand emotional states and fatigue levels	
	Social proximity	Passive devices for establishing the structure and closeness of team members to assess cohesion	
	Virtual reality	Tasks completed in simulated environments to assess behavior, health, and performance	
	Biomarkers	Underlying biological markers that are indicative of stress, resilience, and health and performance	
**Digital countermeasures**			
	Multimedia training (eg, VSS^a^)	Training and education resources provided to individual to help them self-regulate their behavior, health, and performance	
	Virtual reality	Simulated environments that create opportunities for sensory and social stimulation	
**Integrated applications**			
	Digital recommender system (eg, DRIFT^b^)	User monitoring data mined to match countermeasures to contextual demands being encountered	
	Robotic assistance (eg, CIMON^c^)	Artificial emotional support provided by agent in the absence of access to external support	
	Conversational AI^d^ (eg, Ejenta)	Responsive agent able to engage in conversation and provide appropriate responses and suggestions based on the content of the conversation	

^a^VSS: Virtual Space Station.

^b^DRIFT: Digital Regulatory Flexibility Tool.

^c^CIMON: Crew Interactive Mobile Companion.

^d^AI: artificial intelligence.

### Monitoring

Numerous studies have focused on monitoring the health of astronauts and analogous populations [13]. Methods that require active user input include digitized diaries [18], cognition tests [19], and optical stress and fatigue detection systems [20,21]. Due to the additional burden imposed by active input requirements, efforts have focused on developing passive monitoring tools for space. Passive monitoring methods include detecting signs of stress in speech [22,23] and nonverbal behavior [24], wearable devices to monitor sleep [25,26] and team cohesion [27,28], and techniques for the real-time sampling of stress biomarkers [29].

With varying levels of granularity, these systems have the potential to provide time-series data capturing intraindividual variability across markers of behavioral and mental health. At present, such monitoring systems are primarily used to collect research data. However, they could also convey information back to the user (or another decision-maker, eg, a crew commander) to inform timely decisions to prevent decrements in health and well-being and to maintain or improve the function of the astronaut and crew [13]. The utility of monitoring tools, especially those that collect data passively, for capturing dynamics in psychological and social functions is still undetermined. Although metrics derived from passive sensors may offer a degree of objective data, important questions remain about what can be inferred from that data about the subjective state of the person or group being monitored [30].

### Digital Countermeasures

In addition to monitoring, assessment, and diagnosis, several digital countermeasures to prevent deterioration in health and well-being during LDSE missions have been developed. For instance, the Dartmouth PATH program has produced interactive media that provides training on reducing stress, enhancing mood, and resolving conflict [12,31]. Self-directed resources, such as the Expedition Application for Peak Psychological Performance [31] or Virtual Space Station [32], may be part of a repertoire of tools made available during long voyages [33].

Virtual reality (VR) and augmented reality are also promising methods for mitigating some of the demands posed by living and working under conditions of long-term isolation and confinement. For example, projects have explored how VR might be used to manage sensory deprivation [34], provide a platform for interaction and social consistency [10,16], and optimize mental health and well-being in space [35]. Although there has been some initial evaluation of these methods, much more work is needed to determine when, how, and why such countermeasures are effective.

Beyond digital countermeasures, a range of other health and well-being countermeasures for space are being developed and tested, such as mindfulness training courses [36]. For LDSE, these programs may eventually be translated in their entirety or at the very least augmented with some kind of digital element (eg, refresher training) [37].

### Integrated Applications

Advances in monitoring and digital countermeasures create the possibility for closed-loop integrated applications [13]. Our research group is currently exploring how individuals can use a relatively simple, private tool to self-manage their well-being [38]. Equipping individuals to monitor key markers of behavior, health, and performance can guide personalized interventions and optimize well-being to improve functioning in extreme environments like space.

More sophisticated systems include those like the Crew Interactive Mobile Companion (CIMON), a robotic teammate that has flown on the ISS and has supported astronauts in completing work tasks. A stated future aim is to attempt to imbue CIMON (or at least one of its offspring) with some degree of “emotional intelligence” (ie, the capacity to detect and respond to emotional states) so that it might provide effective psychosocial and health and well-being support to astronauts [39]. Similarly, the Translational Research Institute for Space Health has recently funded a project by Ejenta, which aims to develop a conversational artificial intelligence (AI) that could provide emotional support to crew members during LDSE missions. Like CIMON, this work draws upon advances in natural language processing, speech recognition, and machine learning.

Although these integrated projects are in their infancy and may be some way off from being realized in the ways imagined, the trend toward using real-time monitoring data to drive smart, personalized behavioral and mental health solutions is likely to continue. With this level of automation, there is a need to ensure that systems are trustworthy, do not cause inadvertent harm, and positively contribute to their users' well-being.

## Considerations for Design

### Overview

While previous work suggests many positive opportunities, caution is warranted when considering technology-driven mental health management solutions. Using the earlier example of CIMON, several issues were highlighted when the robot was flight-tested on the ISS. For example, it would not turn off on voice command, requiring the astronaut running the experiment to repeat himself. Later, CIMON responded to requests in a way that may be perceived as controlling by the user (eg, after the astronaut asked CIMON to stop playing music, it replied using guilt-inducing language by saying “Don't you like it here with me?”). Although the prior example led to amusement amongst observers, if CIMON was truly relied upon as an integrated member of a deep-space crew, this could lead to problems.

More broadly, and to illustrate this point further, imagine what if instead of promoting positive mental health, systems like VR exacerbated feelings of separation, isolation, and confinement by continually reminding astronauts what they cannot have. Or, if intelligent conversational agents (like CIMON), drawing upon poorly validated monitoring data, suggest an individual coping strategy that undermines crew health and safety. Or, maybe the algorithms underpinning autonomous systems include design and training data biases that make them less useful (or indeed more harmful) to crew members from different cultural, ethnic, or neurodiverse backgrounds. Biases might come in many shapes and forms and range from poor accuracy in emotion recognition (eg, mistaking one emotion for another) and inappropriate attributions of a facial or linguistic expression to a particular internal state (eg, assuming a jovial comment corresponds to an individual being happy) to culturally inappropriate suggestions for stress mitigation (eg, attempting to self-manage an issue where seeking support might be most appropriate).

Clearly, the end goal of these systems is to maintain and improve health and well-being. However, to date, most of the work in this area has been driven by technological opportunity rather than cogent psychological (and broader social science) theory. One issue with such a technology focus, often a theoretical approach to design, is that the human user's diverse needs can be underestimated. Further, in the absence of a guiding theory, if the technology does not influence the psychosocial experience in an expected way, it can be difficult to explain why [40]. Even if the technology does influence the psychosocial experience as expected, absent theorizing about the mechanisms makes it challenging to identify further opportunities and risks or potential boundary conditions.

### Psychological Considerations

Using psychological theory to inform careful design should reduce the likelihood of health technologies having an adverse impact on individuals and crews. Both psychology and human factors research have a rich set of frameworks that could help guide design decisions to prioritize well-being [41]. According to positive computing principles in human factors research, there are 3 ways that digital systems can be designed for well-being [42]. Measures can be preventative, active, or dedicated. The preventative design looks for obstacles to well-being within the system, which, if identified, triggers a redesign. The active design integrates well-being promotive features into an application that is not directly targeted at improving mental health. Dedicated design deals with technologies that are intentionally built for well-being and human flourishing. Although at different stages of maturity, the applied work discussed earlier would benefit from adopting a dedicated design approach. Moreover, all technologies designed for human spaceflight, well-being focused or otherwise, would benefit from taking, at minimum, a preventative approach.

A second issue concerns design for engagement. As astronauts will be responsible for their own self-care and self-management on LDSE missions [9], it is crucial that they feel motivated to use technologies designed to support their well-being. Timely engagement is critical, as interaction with these systems may be most salient precisely when they are psychologically vulnerable (eg, experiencing stress, fatigue, maintaining a high workload, having low motivation, or having interpersonal difficulties) and, thus, less motivated to engage with these systems. Both system engagement and well-being could be promoted by applying coherent psychological frameworks to design. One such approach would be to use a model like the Motivation, Engagement, and Thriving in User Experience (METUX) framework, which encourages designers to consider how technology influences health and well-being at multiple levels of influence. For instance, by considering the impact of interactions with the interface and technology-enabled tasks, and the influence of the technology on behavior and life impact outside of technology use [43]. METUX is theoretically grounded and draws specifically upon tenets within basic psychological needs theory [44], which suggests that human beings function optimally when their needs for autonomy (ie, a sense of volition), competence (ie, a sense of effectiveness), and relatedness (ie, a sense of connection) are met [45].

In support of the METUX model, emerging evidence suggests that consideration of basic psychological needs can foster user engagement [46], enjoyment, and motivation [47], improve learning [48], and contribute to mental health and well-being [49]. In keeping with these findings, recent evidence from Mars isolation and confinement experiments indicates that when basic psychological needs are fulfilled, crew members report better quality motivation and happiness, are less stressed, work more cooperatively, engage in less oppositional defiance, and perform to a higher standard [50]. Such results underline why designing digital technologies with users' basic psychological needs in mind is beneficial for astronaut health, well-being, and function in space.

Although crew members will operate more autonomously during LDSE missions than they do in LEO, this does not imply that they will experience autonomy in the way that the basic psychological needs theory and the METUX models describe [40]. Enforced independence and self-reliance may actually undermine feelings of autonomy and pose risks to competence and relatedness, especially if individuals feel ineffective at managing demands, are understimulated, and feel disconnected from other crew members, or abandoned by those on Earth [1]. Digital health technologies for LDSE could minimize these effects by considering METUX design principles.

**Figure 1:**
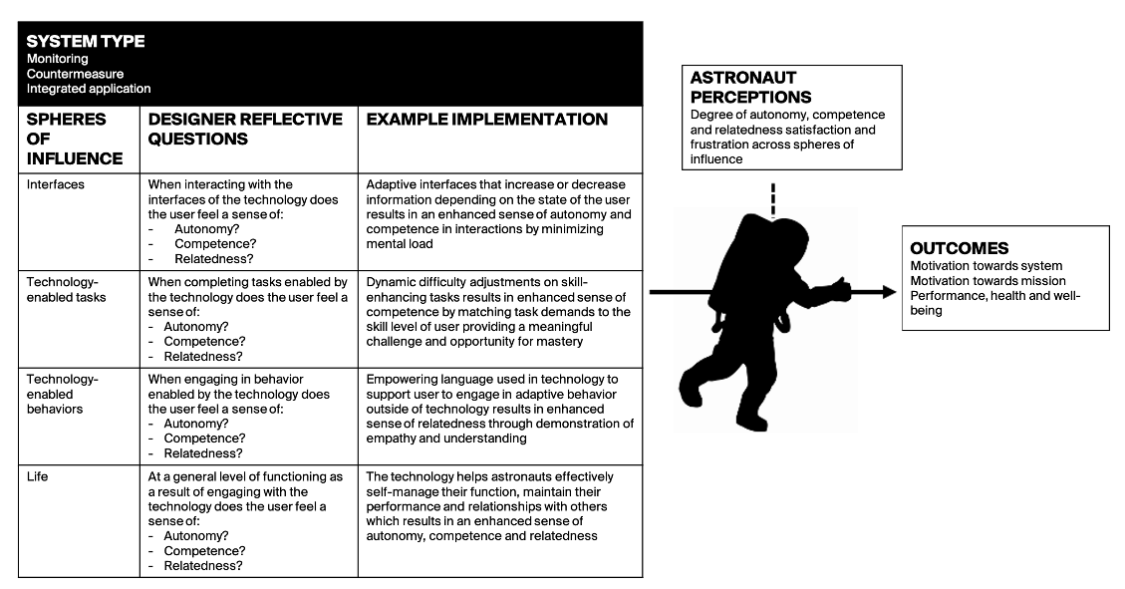
Motivation, Engagement, and Thriving in User Experience (METUX) model applied to human spaceflight.

In Figure 1, we have provided a basic visual guide for designing off-world applications using the METUX principles. In essence, we suggest that the type of digital technology being developed will directly shape design decisions in each of the spheres of influence. At each level, designers are prompted to think about how users experience the technology via the impact the interactions with the technology have upon their basic psychological needs for autonomy, competence, and relatedness. If users (astronauts in this case) experience need fulfillment both in and beyond the technology use, this should result in motivation and engagement in later interactions with the technology and have broader benefits for their behavior, performance, and health.

### Designing for Basic Psychological Need Fulfillment

#### Autonomy

Designing for autonomy means that astronauts' agency, initiative, goals, and values are supported in design. Examples include allowing the user to personalize interfaces, modify information presentation, and make choices regarding task completion [43]. At the same time, designing system adaptability can help avoid autonomy-frustrating experiences. An example might be digital interfaces that adapt to an astronaut’s potential oculomotor and cognitive deterioration during long-duration spaceflight [51].

Autonomy is increased by empowering astronauts with information to self-manage their health. This requires consideration of ways such information is communicated, including written text and speech used by conversational AI and interactive robots. Systems can provide information and guidance in an autonomy-supportive way, considering the user's perspective and providing rationale and clear directions [52]. Alternatively, systems can provide information or recommendations in a controlling manner, applying overt pressure using controlling language and extrinsic rewards. If an astronaut perceives a system they interact with as controlling, this autonomy-frustrating experience may lead to adverse responses, including irritation and oppositional defiance [50].

#### Competence

Features that support an astronaut in feeling successful and effective promote competence in them. For instance, health monitoring systems might include task alert features that direct astronauts’ attention to important information, indicate what the data means, explain what steps they can take to improve their health, and provide positive feedback on effort and progress [53]. This could relieve pressure on an astronaut who may already be operating under high-workload conditions or be fatigued, thus making it easier for them to make competence-enhancing health decisions.

Digital technologies designed to mitigate skill fade through training, whether technical or motor skills (eg, docking simulations) or health-focused (eg, mindfulness or conflict management), could provide personalized, task-focused feedback that allows the user to acknowledge progress, understand how to continue improving, and apply the information learned. Competence may also be enhanced by ensuring adequate novelty in system features, which may counter some of the monotony and boredom risks associated with LDSE missions. This might include, for example, delayed feature releases and diversity in content to maintain interest and engagement. Dynamic difficulty adjustments [47], which consider the user's ability and their current physical and psychological state, can be integrated. For instance, exercise scenarios in a VR application could be made more or less difficult to promote a sense of growing competence.

#### Relatedness

When a crew member feels supported, trusted, and connected to members of their team, mission control, family and friends, and the physical environment, they will experience a sense of relatedness. Digital technologies could support astronaut-relatedness in various ways. At a basic level, computers might be used to deliver training in areas such as conflict management [31] and interacting with empathy and compassion [43], important competencies for relationship management, and thus maintaining a sense of relatedness with others. More advanced systems might be used to monitor when crew members are feeling particularly low in social fulfillment and use innovative approaches for encouraging social interaction with other members of the crew or people at home (eg, VR worlds). Such approaches would have to be carefully managed given the communication delays that are likely to mark LDSE missions and the potential to exacerbate feelings of isolation and loneliness, especially if communications are suddenly disrupted or even terminated. Robotic companions powered by AI may adopt the role of a supportive team member and contribute to relatedness fulfillment by using therapy-based behaviors (eg, attentive listening) [52]. Technologies may also be used to prime astronauts to take time to appreciate the views of space during their journey. Augmented reality programs may induce emotional and cognitive states that allow an individual to feel connected to and reap the restorative benefits of awe-inspiring scenes [54].

#### Evaluation

An important part of designing digital health technologies based on robust and well-evidenced psychological principles, like those included in METUX, is that design decisions can be tested using evidence-based hypotheses. Using already validated psychometric measures (eg, Technology-based Experience of Need Satisfaction; TENS–Interface) [43], it would be possible to test, for example, the impact of interface personalization on experiences of astronaut autonomy, how dynamic difficulty adjustments in VR environments might impact competence fulfillment, or whether supportive language adopted by conversational AI impacts upon relatedness. Those tests can then inform iterative improvements to features in digital systems to ensure that they make a positive contribution to astronauts' behavioral and mental health.

#### Ethical Considerations

Intertwined with the psychological considerations discussed in the prior section are ethical issues related to trust, privacy, and human autonomy. Astronauts have historically been reluctant to talk about psychological difficulties, partly because of the potential consequences for their future mission allocation [55]. Some (anecdotal) evidence indicates, however, that some astronauts welcome the opportunity for private discussions with psychologists [56]. Such discussions may be impossible on LDSE missions, and digital “counselling” technologies may be offered instead. Some astronauts may receive automated health and well-being technologies quite well. In health care settings, patients have reported feeling safer during interactions with automated digital health tools [57]. However, an astronaut’s acceptance of such tools may depend on their confidence in any assurances of confidentiality.

Beyond initial engagement, if astronauts are to continue using such systems, they must also be confident that the information they receive is accurate and helpful. If monitoring data seems invalid or suggested solutions are ineffective, trust in the system will likely decline [58]. We argue that the design of such tools should be based on validated and well-evidenced concepts, including drawing on theories that explain the link between contextual demands and effective coping strategy selection [59], thus avoiding “black box” decisions and allowing astronauts to understand why certain features have been included or specific recommendations made.

Recent critiques of AI systems have highlighted the importance of having robust and representative data sets to avoid biased outcomes that result from biased training data sets. Identifying such data sets for well-being support in space is a particular challenge. Given the small number of individuals who experience LEO spaceflight (let alone LDSE), a representative data set of any significant size will be challenging to collect. Using an unrepresentative data set could result in irrelevant or erroneous recommendations to astronauts that might significantly affect their behavior, health, and performance outcomes. One possible way to mitigate the risk of an unrepresentative data set is by using an initial training data set collected in analogous settings, such as exploration in the polar or other extreme environment settings, commonly viewed as spaceflight analogs.

Privacy risks arising from the use of health surveillance also need to be considered during system design [60]. Designing for privacy can be viewed as a subset of designing for the basic psychological need for autonomy. Features intended to support user empowerment, such as self-tracking, can also pose risks to autonomy if the data collected is shared without consent or used for purposes that are perceived as manipulative or controlling [61]. High-profile missions into deep space will come with intense scrutiny and protecting what little privacy remains will be a high priority for many astronauts. Nevertheless, the design and deployment of health and well-being support systems must also anticipate the possibility of impaired capacity to reason and make decisions as a result of, for instance, cognitive changes or a decline in mental well-being over the course of LDSE missions [14]. Balancing these competing requirements in digital health applications for LSDE will be challenging.

## Future Applications

There are various ways in which this viewpoint might stimulate future research and applied work. The framework and concepts discussed in this paper could be used to inform the design of new digital tools for space. This may be especially pertinent to work that is yet to be augmented (or at least in its early stages) by digital approaches. For example, work on mindfulness training for astronauts [36]. The framework discussed could also be used as a diagnostic for preventative design. If existing digital systems are leading to frustrating user experiences and a lack of engagement, METUX could be used to examine how and why the design is resulting in such experiences and support redesign for more optimal human-computer interactions. Finally, although this study was primarily focused on designing tools for off-world endeavors, most of the systems used to shape the commentary have been developed and tested in space-analog environments on Earth. Thus, we suggest the design principles discussed are equally applicable to the development of well-being supportive technologies for ICE groups on our home planet.

## Conclusions

Astronauts in isolated and confined conditions, experiencing the psychological and physical stressors of LDSE missions, are at risk of deteriorating behavioral and mental health. Digital health technologies have the potential to help manage these risks, but theory and evidence-based design must catch up with the impressive technology-led progress that has been made on tools to monitor and support astronaut health and well-being. Employing robust psychological theory in the design process and carefully considering ethical issues from the outset will increase the likelihood that digital health systems will have positive outcomes, thus making a crucial contribution to the success of future LDSE missions.

## Abbreviations

AI: artificial intelligence
CIMON: Crew Interactive Mobile Companion
ICE: isolated, confined, and extreme
ISS: International Space Station
LDSE: long-duration space exploration
LEO: low Earth orbit
METUX: Motivation, Engagement, and Thriving in User Experience
VR: virtual reality
